# Jorge Lobo’s Disease in Child with Tick Exposure, Brazil

**DOI:** 10.3201/eid3110.250209

**Published:** 2025-10

**Authors:** Franciely Gomes Gonçalves, Gabriel Zorello Laporta, Sebastião Afonso Viana Macedo Neves, Vânia Lúcia Queiroz de Barros, Rosineide Ferreira Bispo, Yally Alves da Silva Sbardelott, Pablo José Custódio Bezerra da Silva, Luiza Alves Vianna, Bernardo Guerra Tenório, João Paulo Romualdo Alarcão Bernardes, Florêncio Figueiredo Cavalcanti Neto, João Nóbrega de Almeida, Marcus de Melo Teixeira

**Affiliations:** Centro Universitário Uninorte (UNINORTE), Rio Branco, Brazil (F.G. Gonçalves); Centro Universitário FMABC, Fundação ABC, Santo André, Brazil (F.G. Gonçalves, G.Z. Laporta); Serviço Estadual de Dermatologia do Acre, Rio Branco (F.G. Gonçalves, V.L.Q. de Barros, R.F. Bispo, Y.A. da S. Sbardelott, P.J.C.B. Sliva); Núcleo de Medicina Tropical, Faculty of Medicine, University of Brasília, Campus Universitário Darcy Ribeiro, Brasília, Brazil (S.A.V.M. Neves, L.A. Vianna, B.G. Tenório, J.P.R.A. Bernardes, M. de Melo Teixeira); Faculdade de Saúde e Medicina, Universidade Católica de Brasília, Taguatinga-DF, Brazil (F.F.C. Neto); Antimicrobial Resistance Institute of São Paulo, São Paulo, Brazil (J. Nóbrega de Almeida Jr); Hospital Israelita Albert Einstein, São Paulo (J. Nóbrega de Almeida Jr, M. de Melo Teixeria)

**Keywords:** Jorge Lobo’s disease, Paracoccidioides lobogeorgii, ticks, vector-borne infections, pediatric mycoses, Amazon Forest, lobomycosis, Acre, fungi, Brazil

## Abstract

Jorge Lobo’s disease (JLD), caused by *Paracoccidioides lobogeorgii*, primarily affects inhabitants of the Amazon Forest. We report a 9-year-old boy in Brazil who had JLD diagnosed after a tick bite. The rarity of pediatric cases likely reflects surveillance gaps. Increased clinical awareness is crucial for early JLD detection and intervention, especially in endemic regions.

Jorge Lobo’s disease (JLD) is a chronic infection caused by the noncultivable fungus *Paracoccidioides lobogeorgii* (previously *Lacazia loboi*) (*1*). JLD primarily affects persons in the Amazon Basin and parts of Central America, and most reported cases originate from Acre state in Brazil (*2*). Infection is thought to occur when skin trauma involving contaminated vegetation or arthropods permits direct fungal inoculation or enables pathogen implantation from secondary environmental sources (*3*). Initial lesions can appear as subtle papules or nodules, often on the ears, arms, or legs, and might evolve into extensive verrucous plaques (*3*,*4*). The hypothesis of environmental transmission is supported by the cessation of new cases after relocation of the Caiabi Indigenous population from endemic to nonendemic areas.

JLD disproportionately affects men involved in rural occupations and forest extractivism. Left undiagnosed, JLD can cause visible disfigurement, functional impairment, and psychosocial stigma, which can undermine the ability to work and can contribute to social isolation. Although few pediatric cases have been documented, the long disease course, which can persist for decades, supports the hypothesis of early-life acquisition and delayed clinical recognition (*4*–*6*). We describe a pediatric male patient with JLD, providing insight into early acquisition and clinical appearance.

In February 2018, a child from a forested area in Brasiléia, Acre state, Brazil, was seen at the Serviço Estadual de Dermatologia do Acre (Rio Branco, Brazil) for a single lesion on his right ear. His mother, who frequently brought him to forest work areas, said that a tick had been attached at the site for 2 days in 2017. Initial treatment with topical dexamethasone (1 mg/g) was ineffective. Over 6 months, the lesion evolved from mild inflammation to a nodular lesion. One year later, a dermatologist suspected JLD on the basis of a solitary indurated lesion on the ear (Figure 1, panel A). Histopathology confirmed the JLD diagnosis, revealing granulomatous inflammation with spherical, double-walled yeast cells in branching chains (Figure 1, panel B) and prominent histiocytes and multinucleated giant cells (Figure 1, panel C).

**Figure 1:**
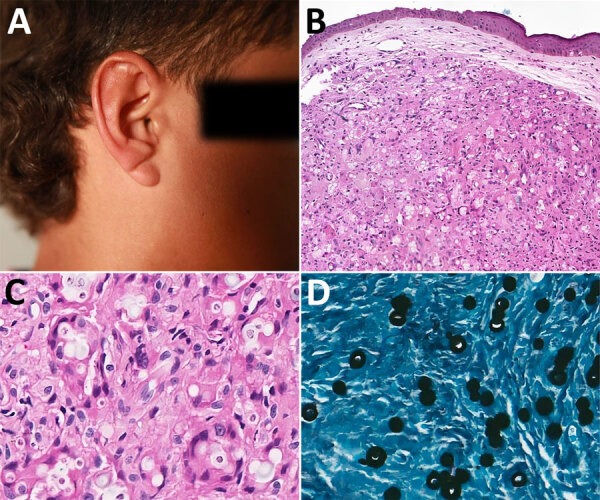
Nodule and histopathology in a case of Jorge Lobo’s disease in child with tick exposure, Brazil. A) Single granulomatous lesion on the right ear, which was surgically excised and histologically examined in 2018. B, C) Hematoxylin and eosin stain of nodule showing cells of the mononuclear phagocyte system, including monocytes, macrophages, epithelioid cells, and multinucleated giant cells, revealing granulomatous inflammation with spherical, double-walled yeast cells in branching chains (B) and prominent histiocytes and multinucleated giant cells (C), which are predominantly dense with areas of mild neovascularization, stromal hyalinization, and a slight infiltrate of lymphocytes and neutrophils. D) Grocott–Gomori methenamine silver stain of recurrent nodule from 2024 in which yeast-like fungal structures are diffusely distributed throughout the stroma and in the cytoplasm of phagocytic cells.

Treatment began with pediatric multidrug therapy for multibacillary leprosy, as previously described (*7*): monthly doses of rifampin (300 mg), clofazimine (150 mg), and dapsone (50 mg), combined with daily dapsone (50 mg) and clofazimine (50 mg on alternate days) for 2 years, and quarterly follow-up visits (*7*). After 4 months, lesion atrophy made surgical excision possible. By November 2020, the patient was lesion-free but was lost to follow-up. In August 2022, during a health outreach mission for leprosy surveillance in the Amazon, the patient was relocated, and a small recurrent lesion on the pinna of the same ear was observed and excised. Itraconazole (200 mg/d) was prescribed for 3 months, but adherence to follow-up consultations was irregular. By June 2024, a 5-cm lesion recurred at the same site. A novel biopsy confirmed JLD recurrence showing chronic granulomatous dermatitis with multinucleated giant cells, fibrotic stroma, and yeast-like cells (Figure 1, panel D). Treatment with itraconazole continued, along with regular medical monitoring.

JLD in children is underreported, although evidence suggests many cases originate in childhood (*8*,*9*). JLD progression, often spanning decades, underscores the need for early detection in endemic regions. Long latency periods support the hypothesis of early-life acquisition (*3*,*4*). The patient’s slow lesion development, despite a year without treatment, further suggests a gradual pediatric course. Timely diagnosis combined with surgical and antifungal therapy can prevent complications (*5*,*10*). JLD elicits a robust inflammatory response with histiocytic infiltrates, multinucleated giant cells, and fungal elements, progressing to fibrosis, chronicity, and immune containment. In this case, a tick bite might have led to fungal entry, as observed in other reports linking JLD onset to arthropod trauma (*3*). Despite several attempts, molecular confirmation (rDNA sequencing) failed because of poorly preserved paraffin-embedded material. Histopathological examination confirmed the diagnosis.

Brasiléia is a municipality in western Acre state, Brazil, bordering Bolivia. It falls under the Brasiléia–Epitaciolândia–Xapuri immediate region (Figure 2), an area marked by rubber plantations and extractive activities, which might increase environmental exposure. Because river transport is seasonal, remote communities face year-round delays in healthcare; floods during the wet season wash out roads and make navigation hazardous, whereas low water in the dry season strands boats and slows travel.

**Figure 2:**
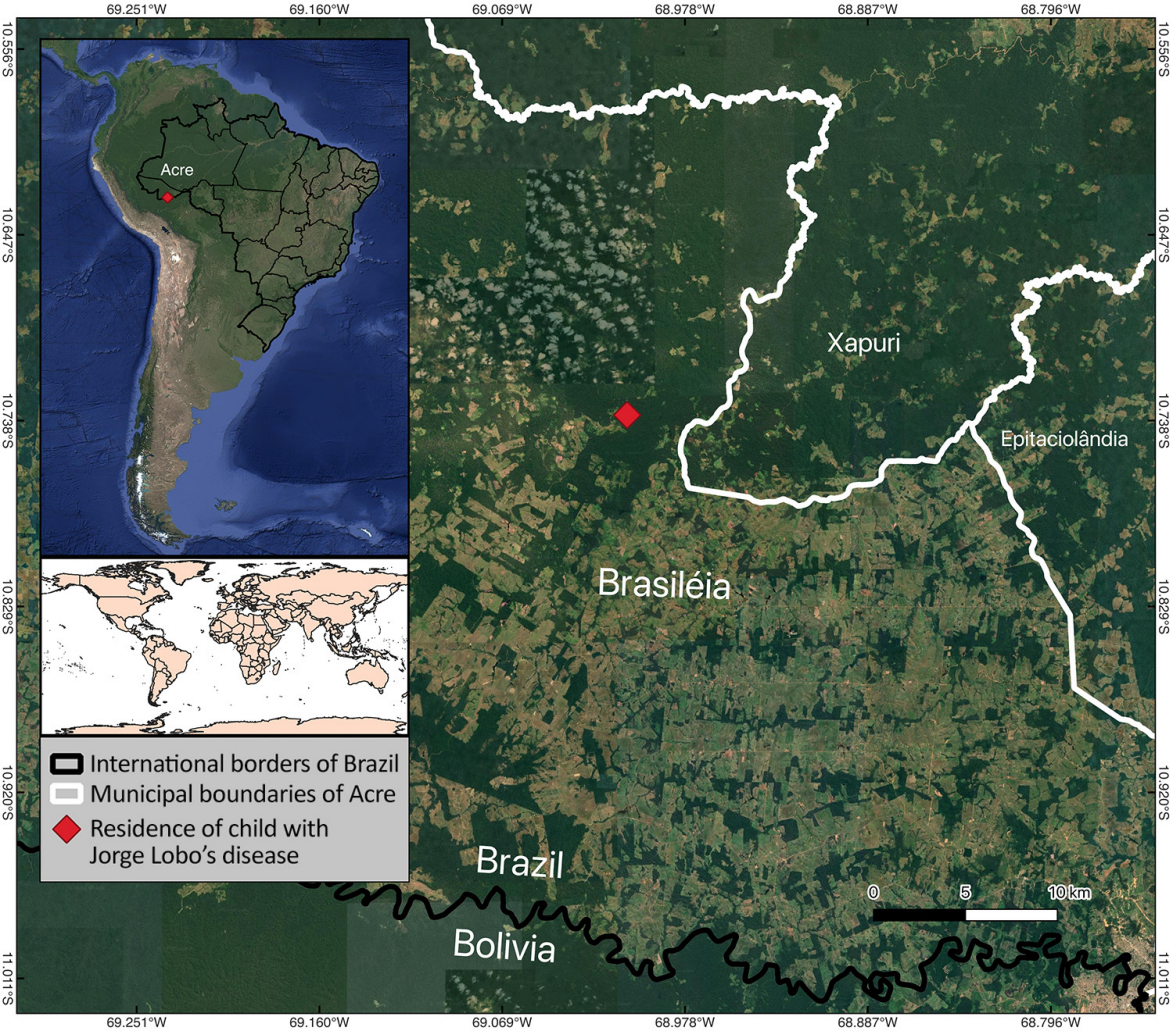
Location of a case of Jorge Lobo’s disease in child with tick exposure, Brazil. The patient resided near the Brazil–Bolivia border and was diagnosed in Brasiléia, Acre state, Brazil. Brasiléia is situated between the municipalities of Xapuri and Epitaciolândia in Acre, also known as Brasiléia–Epitaciolândia–Xapuri immediate region. This zone borders Bolivia within a dense rainforest region of the western Amazon and is marked by rubber plantations and extractive activities.

In summary, treatment for JLD remains empirical, and no standardized protocols are available (*4*,*5*). This case illustrates a 9-year disease course marked by relapses and partial responses and echoes adult reports of forest exposure during childhood, suggesting underdiagnosed childhood infections. Polychemotherapy regimens used for multibacillary leprosy have shown efficacy in localized JLD cases, although prolonged treatment is often necessary (*7*). In this case, therapy lasted 2 years and was complicated by irregular follow-up. Itraconazole regimens in adults typically span 12–24 months (*5*), but data on pediatric treatments are scarce. The patient’s rediscovery during a targeted field mission highlights the need to strengthen active surveillance and outreach in endemic forested areas to enable early detection, timely treatment, and improved management and to mitigate the long-term effect of JLD on at-risk populations.
